# SCH23390 and a humanized anti-cocaine mAb decrease the latency to cocaine-induced reinstatement of lever pressing behavior in rats that self-administer cocaine

**DOI:** 10.1038/s41598-023-41284-1

**Published:** 2023-09-04

**Authors:** Dakota B. Zinani, Jhanvi N. Desai, Andrew B. Norman

**Affiliations:** Department of Pharmacology and Systems Physiology, University of Cincinnati College of Medicine, Cincinnati, OH USA

**Keywords:** Addiction, Addiction

## Abstract

In rats that self-administer cocaine, the latency to the reinstatement of lever pressing behavior induced by a single dose of cocaine is due to the time taken for cocaine levels to fall to the satiety threshold. The D1 dopamine receptor antagonist SCH23390, and the recombinant humanized anti-cocaine mAb h2E2 increase the cocaine satiety threshold and would be expected to alter the latency to reinstatement. Male rats acquired cocaine self-administration behavior on an FR1 schedule. These rats received a single injection of cocaine (12 µmol/kg i.v.) after an i.v. injection of SCH23390 or an infusion of h2E2 or vehicle. The latency to, and the duration of, lever pressing was measured but the presses had no consequence. SCH23390 decreased the latency to lever pressing consistent with dose-dependent increases in satiety threshold. The duration of lever pressing behavior was inversely proportional to the SCH23390 dose suggesting that SCH23390 also increased the cocaine compulsion zone. The mAb h2E2 also produced a similar decrease in latency to responding that gradually reversed over 2 weeks. SCH23390 and h2E2 had an additive effect on the decreased latency to cocaine-induced lever pressing. The single cocaine dose reinstatement paradigm within the context of the compulsion zone theory is a useful pharmacological bioassay system to explore potential pharmacotherapies for relapse prevention in cocaine use disorder.

## Introduction

Lever pressing behavior in rats trained to self-administer cocaine extinguishes shortly after access to cocaine is terminated and can then be reinstated by administration of a single dose of cocaine^1,2^. Single non-contingent doses of several other drugs of abuse including amphetamines and opioids are reported to reinstate/prime responding in animals trained to self-administer the drug^3–8^. The cocaine-induced reinstatement has been suggested to represent a model of persistent relapse in humans with cocaine use disorder^1,2,9–12^.

It has been established that the non-contingent single cocaine dose reinstatement paradigm in rats can be explained in terms of the compulsion zone theory^13^. Rats exhibit lever pressing behavior only when cocaine levels are above the priming/remission threshold^14,15^ and below the satiety threshold^16^. The range of cocaine levels between these thresholds constitute the compulsion zone^17^. Additionally, it has also been established that the number of lever presses following a single cocaine dose is highly variable, and that the latency to reinstatement of lever pressing behavior after the cocaine injection is the most prominent and reproducible dependent measure^13^.

It is well established that dopaminergic neurotransmission plays a prominent role in mediating cocaine self-administration behavior^18–21^. Studies in non-human primates report that several dopamine receptor antagonists dose-dependently decrease the rate of responding after a single injection of cocaine^10,22^, demonstrating that this assay system is sensitive to dopamine receptor antagonists. However, studies of dopamine receptor antagonists on the single dose reinstatement paradigm in rats focus on the number or rate of lever presses as the dependent measure^12^. Changes in the number or rate of lever pressing behavior in rats may not be as reliable a measure of cocaine effects than latency to reinstatement of lever pressing^13^.

The D1 dopamine receptor antagonist SCH23390 is known to affect cocaine self-administration behavior in rats^23–27^, and increase the satiety threshold^25^ and the priming threshold^15^. As the single cocaine dose reinstatement paradigm can be explained in terms of the satiety and priming/remission thresholds^13^, it would be expected that SCH23390 will have measurable effects on latency to reinstatement and the duration of lever pressing. Despite having a different mechanism of action to SCH23390, the recombinant humanized anti-cocaine monoclonal antibody, h2E2, binds to cocaine and acts as a chemical antagonist of cocaine’s effects by sequestering cocaine in the blood. It also increases the priming and satiety thresholds in rats self-administering cocaine^28^, and would also be expected to have effects on latency to reinstatement of lever pressing after a single cocaine dose.

We therefore investigated the effects of these two antagonists on the single dose reinstatement paradigm in rats trained to self-administer cocaine and hypothesize that SCH23390 and h2E2, by raising the cocaine satiety threshold, will result in a decrease in the latency to responding after a single dose of cocaine.

## Results

### SCH23390 decreased the latency to the reinstatement of lever pressing behavior by raising the cocaine satiety threshold

**Figure 1:**
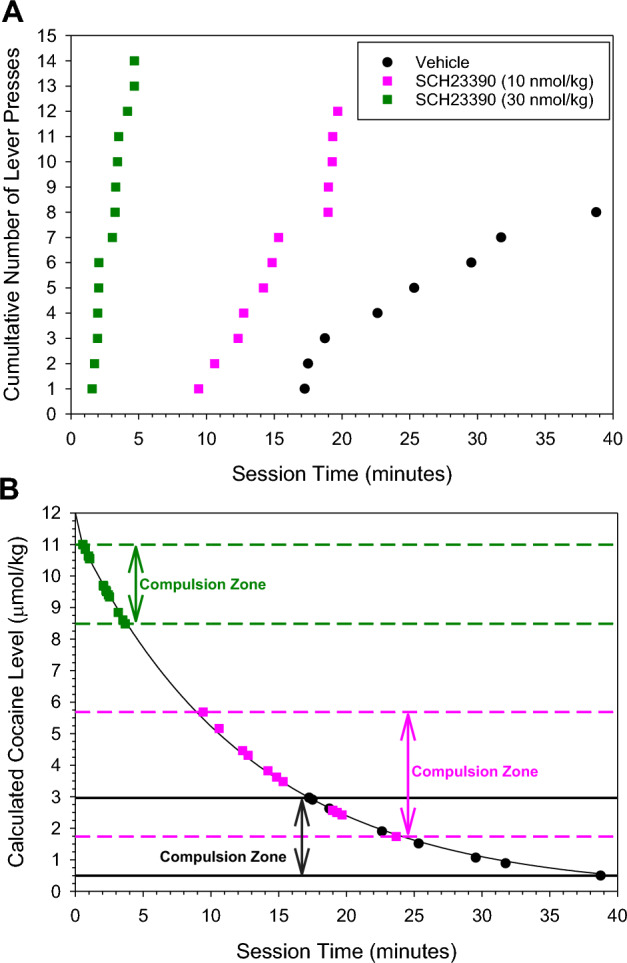
Representative sessions from the same rat showing the cumulative number of lever presses (**A**) and calculated cocaine level at the time of each corresponding lever press (**B**), following a single 12 µmol/kg (i.v.) dose of cocaine administered after an i.v. injection of the vehicle, 10 nmol/kg SCH23390, and 30 nmol/kg SCH23390. Each symbol indicates the time of a lever press. The 20 nmol/kg dose of SCH23390 is not included for clarity. The rat had previously acquired stable cocaine self-administration behavior.

**Figure 2:**
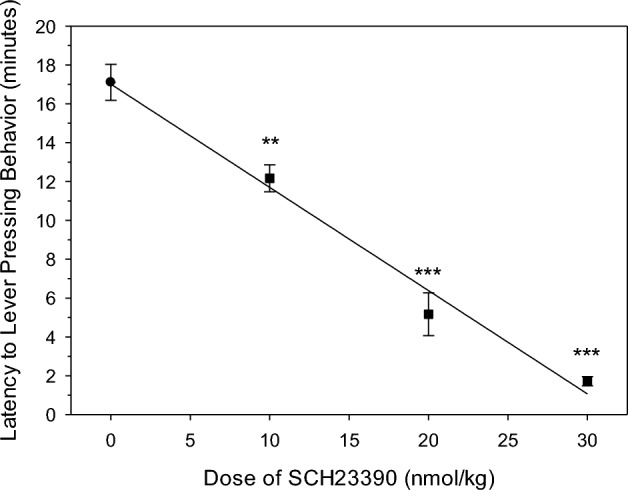
Latency to reinstatement of lever pressing behavior following a single 12 µmol/kg (i.v.) dose of cocaine administered after an i.v. injection of the vehicle (black circle), 10 nmol/kg SCH23390, 20 nmol/kg SCH23390, and 30 nmol/kg SCH23390. Symbols represent mean ± SEM latency. SCH23390 significantly decreased the average latency to reinstatement of lever pressing behavior compared to the vehicle. ** P < 0.01, *** P < 0.001. The total number of rats (N) and sessions run by those rats (n) for each dose 0/10/20/30 nmol/kg of SCH23390 were N = 11 n = 32, N = 8 n = 31, N = 11 n = 14, N = 7 n = 7, respectively.

In the absence of antagonist rats started responding approximately 17 min after the i.v. cocaine injection (Figs. 1A, 2). In the presence of increasing i.v. doses of SCH23390, this latency to the onset of lever pressing was significantly shortened in a dose-dependent manner (Figs. 1A, 2). The magnitude of the decrease in latency was from 17 to 2 min at the 30 nmol/kg SCH23390 dose (Fig. 2). This dramatic decrease was highly reproducible as demonstrated by the relatively small variance (Fig. 2), which was also observed across the other doses.

The shortened latency to the onset of lever pressing in the presence of SCH23390 indicates that responding occurs at higher cocaine levels (Fig. 1B). The calculated cocaine level at the time of the first lever press was dose-dependently higher. Similarly, the cocaine level at the time of the last lever press was also dose-dependently higher.

### SCH23390 decreased the duration of cocaine-induced lever pressing behavior

**Figure 3:**
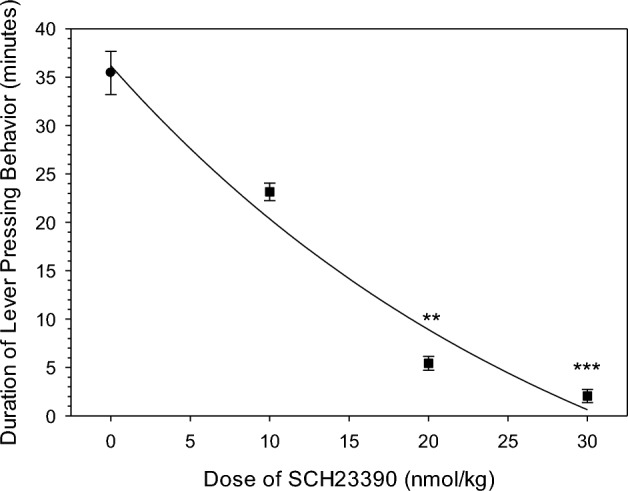
Duration of lever pressing activity following a single 12 µmol/kg (i.v.) dose of cocaine administered after an i.v. injection of the vehicle (black circle), 10 nmol/kg SCH23390, 20 nmol/kg SCH23390, and 30 nmol/kg SCH23390. Symbols represent mean ± SEM duration. SCH23390 significantly decreased the average time of lever pressing activity following single cocaine dose reinstatement compared to the vehicle. ** P < 0.01, *** P < 0.001. The total number of rats (N) and sessions run by those rats (n) for each dose 0/10/20/30 nmol/kg of SCH23390 were N = 11 n = 32, N = 8 n = 31, N = 11 n = 14, N = 7 n = 7, respectively.

The duration of lever pressing behavior was approximately 35 min in the absence of SCH23390 (Fig. 3). In the presence of SCH23390, the duration of lever pressing behavior decreased significantly at 20 and 30 nmol/kg. At the highest dose, the duration of lever pressing behavior was only approximately 2 min (Fig. 3). This was highly reproducible as demonstrated by the relatively small variance (Fig. 3).

### The number of lever presses is an unreliable measure of SCH23390 effects on cocaine-induced reinstatement

In contrast to the dose-dependent decreases in the latency to reinstatement (Fig. 2) and duration of responding (Fig. 3), there was no detectable change in the number of lever presses across these SCH23390 doses despite an apparent decrease from the vehicle values (Supplemental Figure S3). There was considerable between session variability demonstrated by the relatively large error estimates.

### The humanized anti-cocaine mAb h2E2 decreased the latency to the reinstatement of lever pressing behavior

**Figure 4:**
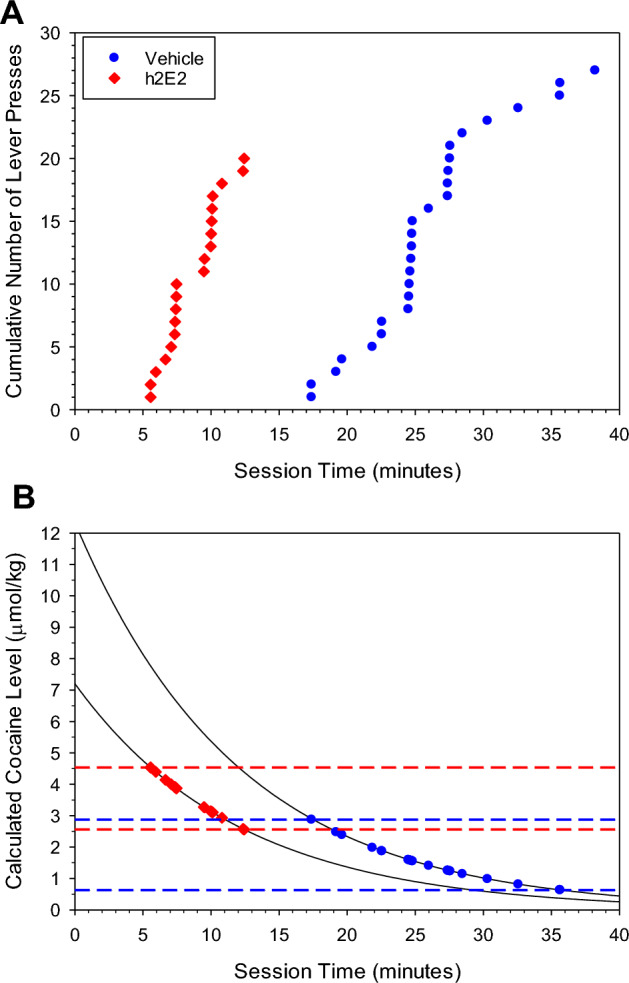
Representative sessions from the same rat showing the cumulative number of lever presses (**A**) and the calculated cocaine level at the time of each corresponding lever press (**B**), following a single 12 µmol/kg (i.v.) dose of cocaine administered after an i.v. injection of the vehicle, or 4.8 µmol/kg dose of h2E2 cocaine binding sites. Each symbol indicates a lever press following cocaine priming. Latency to the onset of lever pressing decreased, and duration of lever pressing activity also decreased in the presence of h2E2 compared to the vehicle. The upper and lower red and blue dashed lines represent the estimated satiety and remission thresholds respectively.

**Figure 5:**
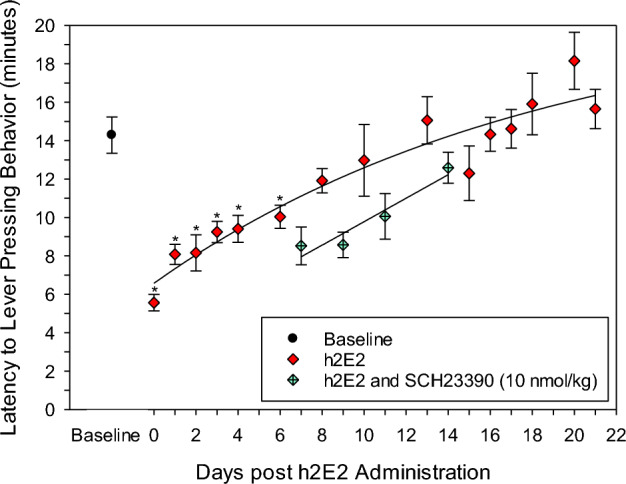
Latency to reinstatement of lever pressing behavior following a single 12 µmol/kg (i.v.) dose of cocaine injection in sessions conducted before and after an infusion of 4.8 µmol/kg h2E2 cocaine binding sites at day 0. Data points represent the Mean ± SEM from 4–7 rats. Significantly different from baseline * P < 0.05. When a 10 nmol/kg dose of SCH23390 was administered before the single cocaine injection, the presence of both h2E2 and SCH23390 further decreased the latency to onset of lever pressing activity.

Prior to the administration of h2E2, there was an approximately 17 min delay to the onset of lever pressing (Fig. 4A), similar to the control session in Fig. 1A. The mean baseline latency for this group of rats was approximately 14–15 min (Fig. 5). After the administration of h2E2, the latency to onset of lever pressing was reduced to approximately 5 min (Fig. 4A). This effect gradually diminished, and over approximately 2 weeks, latency returned to baseline values (Fig. 5).

The effect of a low dose of SCH23390 appeared to be additive to the effects of h2E2 (Fig. 5). The decrease in magnitude of the latency produced by SCH23390 over time could be accounted for by the diminishing effect of h2E2 (Fig. 5).

In the absence of h2E2, the onset of lever pressing behavior occurred when the cocaine level was similar to that observed in the control session in Fig. 1A (Fig. 4B). As illustrated in Fig. 4B, because h2E2 is known to decrease brain cocaine levels, it was assumed that only a fraction of the administered cocaine dose reached the brain and that this could account for the reduced latency to the onset of lever pressing behavior.

## Discussion

As reported previously, the latency to onset of lever pressing behavior was the most prominent observation in the single dose reinstatement method^13^. The single 12 µmol/kg dose of cocaine resulted in an initial calculated cocaine level that was above the satiety threshold, and it took some time for the calculated cocaine levels to decline to the satiety threshold, where rats started lever pressing. Rats maintained lever pressing while cocaine levels fell through the compulsion zone and responding ceased when cocaine levels reached the remission threshold.

It has previously been shown that SCH23390 raises the satiety threshold in rats self-administering cocaine^25–27^. The dose-dependent reduction in latency to lever pressing behavior is consistent with a SCH23390-induced increase in cocaine satiety threshold as it takes less time for the cocaine level to fall down to the elevated satiety threshold (Fig. 1B).

As SCH2339 increases both the satiety threshold and the priming threshold, the upper and lower limits of the compulsion zone, it is expected that the cocaine compulsion zone should have also been raised. This is consistent with the decreased duration of lever pressing activity as a function of SCH23390 dose because of the first order elimination of cocaine. As cocaine elimination rate is proportional to its concentration, cocaine levels would remain in the compulsion zone for a shorter duration at the higher cocaine levels.

It is established that SCH23390 reliably produces catalepsy in rats^29^. However, in the present studies, the latency to the induction of responding was decreased. If cocaine-induced locomotion was impaired, latency would be expected to increase. This is consistent with previous reports that SCH23390 does not produce a general impairment of responding in rats self-administering cocaine^23^. The single dose reinstatement paradigm may be another useful method for assessing interactions between stimulants and dopamine receptor antagonists.

Similar to the effect of SCH23390, h2E2 also decreased the latency to onset of lever pressing behavior in these rats (Figs. 1A, 4A). However, h2E2 and SCH23390 have distinct mechanism of action. SCH23390 is a competitive antagonist of D1 dopamine receptors while h2E2 binds directly to cocaine in the peripheral circulation^30^, acting as a chemical antagonist of cocaine’s effects. The mAb h2E2 also modestly raised the priming threshold during cocaine self-administration in rats^28^. The 4.8 µmol/kg h2E2 dose of cocaine binding sites could theoretically reduce the apparent cocaine concentration produced by the 12 µmol/kg dose to approximately 7.2 µmol/kg (Fig. 4B). If so, the reduction in latency would be due to an apparently lower cocaine dose reaching the satiety threshold in the brain sooner. This would be consistent with the cocaine dose-dependency of latency to onset of lever pressing behavior in rats^13^. It appears that the 4.8 µmol/kg h2E2 dose of cocaine binding sites is approximately equieffective to a 20 nmol/kg SCH23390 dose (Figs. 2, 5). So, it appears that h2E2 is less potent than SCH23390. This demonstrates how latency to responding with the single cocaine dose reinstatement method can be used as a pharmacological assay system.

Although the assumption that h2E2 sequestered 4.8 µmol/kg of cocaine suggested that the cocaine satiety threshold increased (Fig. 4B), h2E2 should have no central effects and it is possible that the satiety threshold was unaltered in the brain. If so then the effects of h2E2 on brain cocaine concentrations were of a greater magnitude than predictions based on cocaine:h2E2 cocaine binding site stoichiometry (Supplemental Figure S1).

The gradual return of latency back to baseline over 2 weeks after a single h2E2 injection (Fig. 5) is consistent with previous studies of the duration of effect of cocaine priming threshold^14,15^ and on the pharmacokinetics of h2E2 in rats and mice^30,31^.

As SCH23390 is structurally different from cocaine, it is unlikely to bind to and interact with h2E2. Therefore, it may be expected that SCH23390 and h2E2 would at least have additive effects on cocaine-induced lever pressing. Indeed, when SCH23390 was administered after h2E2, an additive effect on reduction of latency was observed. The decline in the effect on latency over time with each subsequent SCH23390 administration can be accounted for by the decline in the effect of h2E2. This demonstrates the feasibility of a combined pharmacotherapy for cocaine use disorder that uses competitive dopamine receptor antagonists in patients potentially treated with h2E2.

The duration of lever pressing in presence of h2E2 (Supplemental Figure S2), was notably more variable than was observed with SCH23390 (Fig. 3), but the significance of this difference in variability is not clear at present.

While the number of lever presses is a popular measure in the single cocaine dose paradigm, it has been previously established that the number of lever presses following a single cocaine dose is highly variable and has weak dose-dependency with the given single dose of cocaine^13^. Similarly, while the number of lever presses decreased in the presence of SCH23390 compared to the vehicle, lever pressing was variable and not dependent on the dose of SCH23390 (Supplemental Figure S3). Additionally, the number of lever presses was highly variable after administration of h2E2 (Supplemental Figure S4). This variability precluded the detection of h2E2-induced changes, in contrast to the clear effects on reduced latency to lever pressing behavior. This emphasizes that the latency to lever pressing behavior is a more appropriate measure in the single cocaine dose paradigm. The time taken to commence responding after the single cocaine dose indicates the duration when cocaine levels are above the satiety threshold. The first lever press indicates the transition of cocaine levels from the satiety zone to the compulsion zone where cocaine-induced lever presses occur. It has been observed that lever pressing behavior in the compulsion zone is variable^13^.

Although this study focuses on the effects of cocaine antagonists on the single cocaine dose reinstatement paradigm in the context of the compulsion zone theory, there are other highly relevant and influential theories of responses to cocaine and dopaminergic neurotransmission. According to the “incentive sensitization theory”, repeated drug use sensitizes only the neural systems that mediate the motivational process of incentive salience (wanting), but not neural systems that mediate the pleasurable effects of drugs (liking)^32^. Therefore, the dopamine receptor antagonist should be expected to antagonize both processes whether sensitized or normal. This would be expected to increase the latency to responding to a priming dose of cocaine. Similarly, according to the “anhedonia hypothesis”, the most subtle and interesting effect of neuroleptics is a selective attenuation of motivational arousal^33^. By decreasing the motivation to obtain cocaine, antagonists should also increase the latency to responding to a priming dose of cocaine. The current results are in contrast to the logical predictions of both the “incentive sensitization theory” and the “anhedonia hypothesis”. An alternative framework for understanding the effects of drugs on dopamine neurons is the “dopamine prediction-error signal hypothesis” that identifies signals for subjective reward value and formal economic utility^34^. However, it is not clear what this theory predicts about the effects of cocaine antagonists on the single cocaine dose reinstatement paradigm.

In conclusion, the single cocaine dose reinstatement method within the context of the satiety threshold theory is a useful pharmacological bioassay system to study the effects of dopamine receptor antagonists on systems underlying cocaine-induced lever pressing behavior. If this behavior is relevant to cocaine use disorder in humans, these antagonist effects may predict therapeutic efficacy. This method works with a range of classes on antagonists, and the latency to onset of lever pressing is the most reliable measure that adds to the utility of this assay system.

## Methods

### Animals

Male Sprague–Dawley rats weighing between 200 and 500 g over the course of the study were purchased from Harlan Laboratories (Indianapolis, IN). Rats were housed individually on a 14/10-h light/dark cycle with unrestricted access to food and water. All studies were conducted in accordance with the National Institutes of Health Guide for the Care and Use of Laboratory Animals and under a protocol approved by the Institutional Animal Care and Use Committee (IACUC) at the University of Cincinnati and reported in accordance with ARRIVE guidelines.

### Catheter implantation

All surgeries were performed using aseptic technique with materials being prior autoclaved or sterilized with vaporized H_2_O_2_. Rats were anesthetized with inhaled isoflurane (RRID:SCR_018956), then the right jugular vein was catheterized with a polyurethane rounded tip, this catheter was connected to the pin of an Instech Vascular Access Button, which was tested for patency by flushing sterile saline through before implantation. Buprenorphine (0.03 mg s.c.) (RRID: AB_10972407) was administered post-surgery for pain control and gentamycin (25 mg s.c.) for three days was used to prevent infection following surgery. Three days following surgeries, catheters were flushed with 100 units/ml of heparin in bacteriostatic saline solution, to extend the patency of the catheters. Following failure of patency (tested using brevital sodium), this process was repeated on the left jugular vein, the left femoral, and the right femoral vein with each subsequent loss of patency throughout the course of the study. Following the loss of the 4th vein patency animals would be euthanized according to IACUC protocols.

### Self-administration training

Detailed protocols for cocaine self-administration training and real time computation of cocaine level were reported previously^35^. In brief, beginning at least five days after surgery, rats were trained to self-administer cocaine HCl. Rats were weighed immediately prior to each self-administration session. Self-administration sessions began from 9 to 10 AM. Animals were placed in isolated chambers containing an active and an inactive lever. During training, a unit dose of 3 μmol/kg (1 mg/kg) of cocaine HCl (supplied by the NIDA Drug Supply Program from the Research Triangle Institute) was delivered on a fixed-ratio 1 (FR1) schedule with a 5 s timeout period or equal to the injection time, whichever was longer. A cue light was illuminated for the duration of the timeout. Rats had access to cocaine for 3 h a day, five days a week. The training was considered complete when inter-injection intervals did not systematically deviate from day to day for three consecutive days.

Every Monday throughout the course of the study, rats were run on a FR1 cocaine self-administration session. The first two self-administered doses were 3 µmol/kg to accelerate the initial loading phase. Then, the next 75 doses were 0.3 µmol/kg and the next 15 doses were 3 µmol/kg. After access to cocaine was terminated, lever presses were recorded until 30 min of no activity and these lever presses had no consequences. These sessions ensured that these rats still self-administered cocaine despite the subsequent days of only receiving a single dose of cocaine.

### Single cocaine dose reinstatement of lever pressing

Following cocaine self-administration training at different unit doses, rats were then switched to single cocaine dose reinstatement session. Single dose reinstatement sessions were run Tuesday-Friday, after placing the rat into the same chambers they were administered a single i.v. 12 µmol/kg dose of cocaine. Lever presses were recorded but had no consequences. Sessions were terminated when 30 min had elapsed since the last lever press. The rats were then returned to their home cages until the next daily session.

### Calculation of cocaine level

The cocaine level at the time of each lever press was calculated as described previously^35^. Briefly, the cocaine level was calculated every second by a non-compartmental pharmacokinetic model assuming first-order elimination with a half-life of 500 s.

### Administration of SCH23390

The saline vehicle or SCH23390 (10, 20 or 30 nmol/kg i.v.) was injected and flushed with 0.1 mL saline immediately prior to placing the rats in the self-administration chambers where a single i.v. 12 µmol/kg cocaine dose was administered. All lever presses following the cocaine infusion were recorded but had no consequences.

### Administration of h2E2

Rats established a baseline behavior several days before the administration of h2E2. Rats were administered a dose of 4.8 µmol/kg dose of h2E2 cocaine binding sites (360 mg/kg h2E2), then returned to their home cage where they stayed for one hour. Following an hour, they were placed into self-administration chambers where they were administered a single dose of 12 µmol/kg cocaine HCl solution (i.v.). Lever pressing behavior was recorded until 30 consecutive minutes of no lever pressing occurred. Sessions were run for up to 21 days after the infusion of h2E2.

### Administration of SCH23390 following h2E2

Past studies have reported the t_1/2_ for h2E2 in rats to be approximately 8–12 days^30,31,36^. Therefore, seven days after administration of h2E2, a 10 nmol/kg dose of SCH23390 was administered before the beginning of the 12 µmol/kg single cocaine dose reinstatement session began. The dose was administered approximately every other day for four sessions.

### Statistical analysis

The parameters measured were the latency to the onset of activity, the duration of activity, the calculated cocaine level at the onset of activity (the satiety threshold), and the calculated cocaine level at the cessation of activity (the remission threshold). These data were collected from multiple rats over multiple sessions, however, as rats lost catheter patency the overall number of animals used fluctuated. All the parameters were analyzed using SigmaPlot 14.5. ANOVA was conducted to compare SCH23390 doses and statistical significance was determined by comparing the values to the vehicle.

Following administration of h2E2 the same parameters were collected each day for 21 days, and statistical significance of these values were compared to the baseline using a One Way ANOVA. Baseline values were collapsed across days. The mean of 3–5 sessions for each of the 7 rats was calculated. These values were averaged. The ANOVA was conducted using SigmaPlot 14.5.

### Supplementary Information

Supplementary Information.

## Data Availability

The data that supports the findings of this study are available from the corresponding author on reasonable request.

## Abbreviation

mAb: Monoclonal antibody
