# Supplementary material for: SCH23390 and a humanized anti-cocaine mAb decrease the latency to cocaine-induced reinstatement of lever pressing behavior in rats that self-administer cocaine
Source: Sci Rep. 2023 Sep 4;13:14566. doi: 10.1038/s41598-023-41284-1 (PMC10477340; doi:10.1038/s41598-023-41284-1)
Supplement: Supplementary file 1 — Supplementary Information. [file 41598_2023_41284_MOESM1_ESM.docx]

**SUPPLEMENTAL INFORMATION**

Supplemental Figure S1. Alternative interpretation of the representative sessions shown in Figure 4. Panel A is the same as Figure 4A. In Panel B, the h2E2 session is plotted assuming that the first lever press occurred at the same cocaine level as the first lever press in the vehicle session. Latency time to the onset of lever pressing decreased, and duration of lever pressing activity also decreased in the presence of h2E2 compared to the vehicle. The upper blue line represents the estimated satiety threshold, and lower red and blue dashed lines represent the estimated remission thresholds.

**
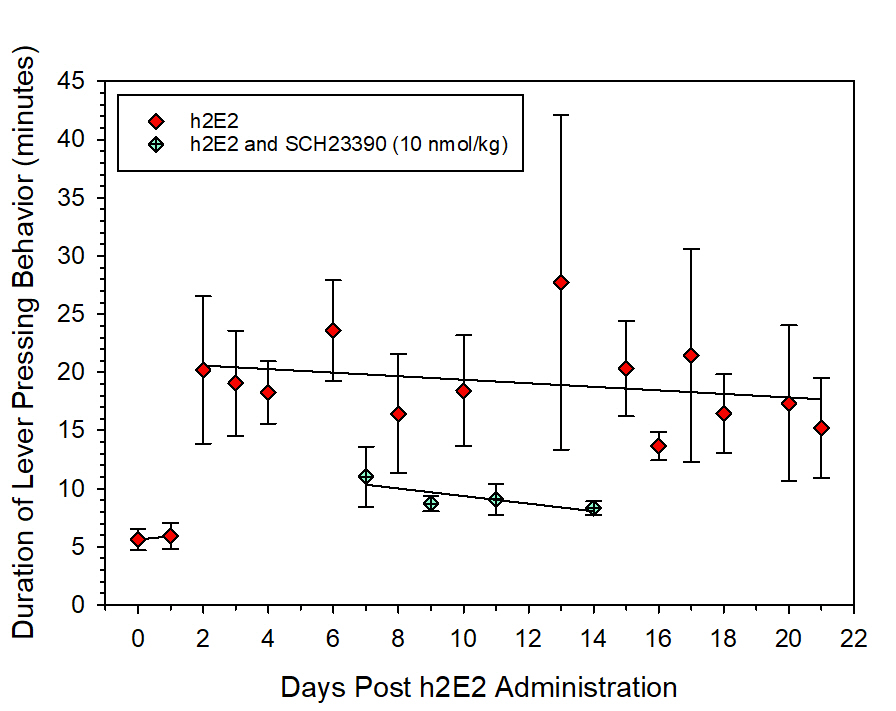
**

Supplemental Figure S2: Duration of lever pressing behavior following a single 12 µmol/kg dose of cocaine injection in sessions conducted over days after an infusion of 360 mg/kg h2E2 at day 0. Data points represent the Mean ± SEM from 4-7 rats. In the presence of h2E2, lever pressing activity averaged at 5.8 minutes for the first two days, increased at Day 2 and remained elevated all the way through Day 21 (average duration 19.1 minutes). When a 10 nmol/kg of SCH23390 was administered before the single cocaine injection, the presence of both h2E2 and SCH23390 decreased the duration of lever pressing activity to an average of 9.28 minutes.

Supplemental Figure S3: The number of lever presses following a single 12 µmol/kg dose of cocaine administered after an injection of the vehicle (black circle), 10 nmol/kg SCH23390, 20 nmol/kg SCH23390 and 30 nmol/kg SCH23390. Symbols represent mean ± SEM number of lever presses. The total number of rats and sessions run by those rats for each dose of SCH23390 is the same as in Figure 2 and Figure 3.

Supplemental Figure S4: Number of lever presses following a single 12 µmol/kg dose of cocaine injection in sessions conducted over days after an infusion of 360 mg/kg h2E2 at day 0. Data points represent the Mean ± SEM from 4-7 rats. Lever pressing activity was highly variable. When a 10 nmol/kg of SCH23390 was administered before the single cocaine injection, the presence of both h2E2 and SCH23390 caused a decrease in the number of lever presses.

| Supplemental Table 2. The time (minutes) of each lever press in the representative sessions shown in Figure 4 | |
| --- | --- |
|  |  |
|  |  |
| Vehicle | h2E2 |
| 17.38 | 5.555 |
| 17.38 | 5.555 |
| 19.18666667 | 5.94 |
| 19.61833333 | 6.65 |
| 21.85833333 | 7.07 |
| 22.54666667 | 7.331666667 |
| 22.56666667 | 7.36 |
| 24.50333333 | 7.401666667 |
| 24.55166667 | 7.445 |
| 24.58833333 | 7.461666667 |
| 24.64166667 | 9.471666667 |
| 24.71 | 9.516666667 |
| 24.75333333 | 9.975 |
| 24.775 | 10.005 |
| 24.81166667 | 10.045 |
| 26 | 10.08 |
| 27.395 | 10.11666667 |
| 27.39666667 | 10.79833333 |
| 27.44166667 | 12.345 |
| 27.54666667 | 12.42666667 |
| 27.58 |  |
| 28.47666667 |  |
| 30.315 |  |
| 32.58333333 |  |
| 35.63166667 |  |
| 35.66333333 |  |
| 38.21833333 |  |
|  |  |

| Supplemental Table 1. The time (minutes) of each lever press in the representative sessions shown in Figure 1 | | |
| --- | --- | --- |
|  |  |  |
|  |  |  |
| Vehicle | SCH23390 (10 nmol/kg) | SCH23390 (30 nmol/kg) |
| 17.24666667 | 9.418333333 | 1.556666667 |
| 17.495 | 10.59666667 | 1.728333333 |
| 18.72833333 | 12.335 | 1.955 |
| 22.625 | 12.74166667 | 1.961666667 |
| 25.33333333 | 14.195 | 2.041666667 |
| 29.545 | 14.835 | 2.048333333 |
| 31.74666667 | 15.32 | 3.051666667 |
| 38.76 | 18.97 | 3.248333333 |
|  | 18.98666667 | 3.3 |
|  | 19.28 | 3.436666667 |
|  | 19.31 | 3.515 |
|  | 19.675 | 4.163333333 |
|  |  | 4.671666667 |
|  |  | 4.678333333 |
|  |  |  |
